# Maternal Psychological Distress and Repeated Implantation Failure: A Mini Review: Short title: Maternal Distress and Implantation Failure

**DOI:** 10.31661/gmj.v15i.4201

**Published:** 2026-06-19

**Authors:** Sina Vakili, Bahia Namavar Jahromi, Sepide Goharitaban

**Affiliations:** ^1^ Infertility Research Center, Shiraz University of Medical Sciences, Shiraz, Iran; ^2^ Department of OB-GYN, Infertility and IVF Division, Shiraz School of Medicine, Shiraz University of Medical Sciences, Shiraz, Iran; ^3^ Department of Anatomical Sciences, Faculty of Medicine, Hamedan University of Medical Science, Hamedan, Iran

**Keywords:** Repeated Implantation Failure, Infertility, Stress, sychological Factors, Fertility Treatment

## Abstract

Repeated implantation failure (RIF) is one of the most stressful and distressing experiences for women undergoing assisted reproductive technologies (ART) [1]. Despite progress in identifying physical and physiological factors affecting RIF, such as embryo selection, endometrial preparation, and laboratory techniques, there has not been much progress in understanding the psychological factors associated with RIF in infertile women. Compared with a single failed ART cycle, RIF is associated with repeated in vitro fertilization (IVF) failures, and repeated cycles of hope and disappointment exacerbate anxiety, depression, and emotional exhaustion in patients [2] (Table-1). According to theories by scientists such as Sigmund Freud and Wilson [3], psychological trauma, including fear of pregnancy, is considered a cause of female infertility. Psychological stress in women with RIF leads to endocrine dysfunction and increased endometrial contractions, negatively affecting IVF success rates and embryo implantation rates [4]. Additionally, women with RIF face psychological stresses such as delayed childbearing age and various financial, social, and family problems during different stages of treatment (before starting, during, and after treatment failure) [5]. These stresses contribute to feelings of hopelessness, decreased self-confidence, marital problems, and the end of relationships [6] (Figure-1 A-C) . Therefore, this review aimed to stimulate reflection on the psychosocial burden of RIF in infertile couples undergoing ART, explore when distress is most intense, consider contributing factors, and discuss implications and gaps for clinical practice and future research.

## Introduction

**Table:** Table1. Definitions of Psychological Concepts

Term	Definition
Trauma	"Any disturbing experience that results in significant fear, helplessness, dissociation, confusion, or other disruptive feelings intense enough to have a long-lasting negative effect on a person’s attitudes, behavior, and other aspects of functioning" [7].
Anxiety	"An emotion characterized by apprehension and somatic symptoms of tension in which an individual anticipates impending danger, catastrophe, or misfortune"[7].
Stress	"The physiological or psychological response to internal or external stressors. Stress involves changes affecting nearly every system of the body, influencing how people feel and behave" [7].
Depression	1. "A negative affective state, ranging from unhappiness and discontent to an extreme feeling of sadness, pessimism, and despondency, that interferes with daily life. Various physical, cognitive, and social changes also tend to co-occur, including altered eating or sleeping habits, lack of energy or motivation, difficulty concentrating or making decisions, and withdrawal from social activities. It is symptomatic of a number of mental health disorders" [7]. 2.
Hopelessness	3. "The feeling that one will not experience positive emotions or an improvement in one’s condition. Hopelessness is common in severe major depressive episodes and other depressive disorders and is often implicated in suicides and attempted suicides " [7].

Note: All of the definitions were directly from the APA Dictionary of Psychology (American Psychological Association, 2026).

**Figure-1:**
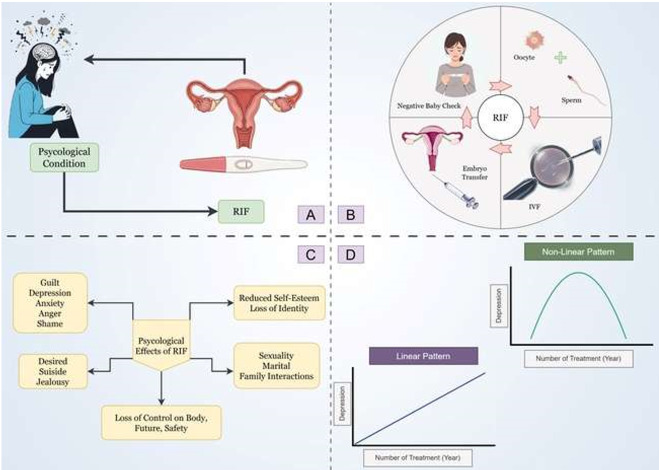
Figure 1. Association of repeated implantation failure (RIF) and psychological conditions. (A) There is a two-way interaction between RIF and a woman's psychological conditions; in that psychological distress can increase infertility rate, and treatment failures exacerbate psychological outcomes such as anxiety and depression. (B) RIF means that embryos do not successfully implant in the uterine wall after several embryo transfers during in vitro fertilization (IVF) treatment. (C) RIF affects psychological functioning across emotional, social, family, and financial domains. (D) Depression and the number of treatment failures in RIF women have two patterns. In the linear pattern, depression increases proportionally with each treatment failure. In contrast, the nonlinear pattern is marked by initially low depression levels that rise with repeated failures but subsequently decline if treatment continues.

**Figure-2:**
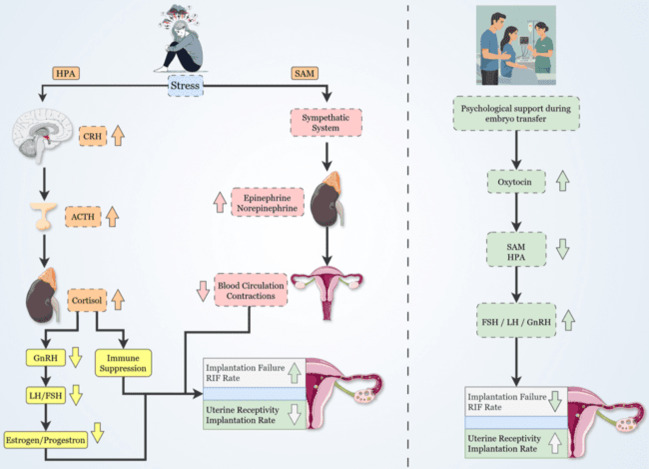
Figure 2. Relationship between cortisol/oxytocin and implantation failure. Stress in infertile women activates the HPA and SAM axes. In the HPA axis, increased CRH/ACTH secretion leads to increased cortisol levels. Increased cortisol, by inhibiting the hypothalamic-pituitary-gonadal (HPG) axis, reduces GnRH secretion and subsequently LH/FSH, thereby lowering sex hormone (estrogen/progesterone) levels. At the same time, activation of the SAM axis increases epinephrine and norepinephrine, which reduce endometrial receptivity and implantation rates. Psychological support during embryo transfer helps improve endometrial receptivity and implantation rates by increasing oxytocin and decreasing SAM/HPA axis activity.

Repeated implantation failure (RIF) is one of the most stressful and distressing experiences for women undergoing assisted reproductive technologies (ART) [1]. Despite progress in identifying physical and physiological factors affecting RIF, such as embryo selection, endometrial preparation, and laboratory techniques, there has not been much progress in understanding the psychological factors associated with RIF in infertile women. Compared with a single failed ART cycle, RIF is associated with repeated in vitro fertilization (IVF) failures, and repeated cycles of hope and disappointment exacerbate anxiety, depression, and emotional exhaustion in patients [2] (Table-1). According to theories by scientists such as Sigmund Freud and Wilson [3], psychological trauma, including fear of pregnancy, is considered a cause of female infertility. Psychological stress in women with RIF leads to endocrine dysfunction and increased endometrial contractions, negatively affecting IVF success rates and embryo implantation rates [4]. Additionally, women with RIF face psychological stresses such as delayed childbearing age and various financial, social, and family problems during different stages of treatment (before starting, during, and after treatment failure) [5]. These stresses contribute to feelings of hopelessness, decreased self-confidence, marital problems, and the end of relationships [6] (Figure-1 A-C) . Therefore, this review aimed to stimulate reflection on the psychosocial burden of RIF in infertile couples undergoing ART, explore when distress is most intense, consider contributing factors, and discuss implications and gaps for clinical practice and future research.

## Definition, Prevalence, and Etiology of Repeated Implantation Failure

Repeated implantation failure (RIF) is a condition in which the embryo fails to implant in the uterine wall despite multiple embryo transfers during IVF treatment [8] (Figure-1B). Different IVF centers propose varying definitions of RIF. For example, some sources define RIF as the failure of embryo implantation after three consecutive cycles, with up to three high-quality embryos transferred in each cycle [9][10]. According to another definition, the absence of a gestational sac approximately 45 days after the transfer of at least 3 embryos, or after the transfer of more than 10 embryos over multiple transfers, is considered RIF [11]. Approximately 20% of couples undergoing ART are RIF patients, who experience significant clinical and psychosocial challenges [12].

Based on a general classification, RIF is categorized into three main types: endometrial, idiopathic, and multifactorial [13][14]. This condition may result from fetal abnormalities, reduced uterine receptivity, uterine anomalies, and the mother’s health status [15][16]. Several factors may influence RIF, including chromosomal and uterine abnormalities, hormonal and placental disorders, smoking, certain medications, maternal heart and kidney disease, and embryo quality [17][18]. In addition to physical and physiological factors involved in RIF, psychological stressors and multidimensional pressures are recognized as influential factors [19].

## Psychological Effects of Repeated Implantation Failure

The diagnosis of infertility often leads to significant psychological and emotional stress for couples. Infertile couples have emotional reactions at the start of ART treatments, but how positive or negative these feelings stay is directly linked to the treatment outcome [20][21]. Since RIF patients undergo repeated treatment cycles, their emotional swings,such as hope and disappointment, happiness and sadness,are described as feeling like an "emotional roller coaster" [22]. Couples who become pregnant after IVF have different emotional reactions compared to those who experience a natural pregnancy and may need more emotional support, especially in the early stages [23].

Decisions like postponing childbearing have contributed to infertility among RIF patients [19]. They may be associated with significant psychological consequences, including increased feelings of shame and guilt regarding past life choices [24][25]. As these emotions continue, the longing for the motherhood experience increases in infertile women [19]. Women experience emotions like jealousy and embarrassment when observing social situations such as the birth of babies, mother-child interactions, and other women's pregnancies [26][27]. Further, because of a lack of mutual understanding from family and friends, they prefer suicide over suffering [28][29].

In infertile couples, women experience more psychological distress than men. Regardless of which couple is infertile, women undergo highly invasive procedures and strict treatment regimens during ART treatment [19]. Furthermore, in the cultures of many societies, women are blamed as the main cause of infertility [30]. In today's world, women try to increase their chances of fertility through various methods such as herbal teas, yoga, energy therapy, prayer, and lifestyle changes [31]. Enduring these hardships and repeated treatment failures creates a sense of failure, which is responsible for infertility and self-blame in RIf women [19].

Infertility, like HIV, is an invisible disability because it cannot be seen and can remain private [32]. Because of this, questions, jokes, and a lack of empathy from others might worsen psychological suffering and lead to reproductive trauma for an infertile person [33][32]. Women who have experienced a single failed treatment report that the wait after embryo transfer is the most stressful part of the process [34][35]. Research indicates that the psychological trauma from this stress can last for up to 20 years [36]. Women with RIF often experience significant anxiety and stress after multiple unsuccessful attempts [12].

RIF women face traumas such as distress, depression, disappointment, and stress, along with a reduced quality of life, because treatment failure is not a single event, but the result of a long and burdensome treatment journey. This journey includes repeated medical and genetic interventions, hormonal stimulation, ultrasounds, embryo preparation and transfer, and a waiting period for results [37][38].

RIF couples may also experience a loss of identity and feel as if their bodies are fragmented [39][40]. These individuals often feel a stronger sense of betrayal for two main reasons. First, they see infertility as their body betraying them. Second, undergoing multiple medical treatments can cause them to feel like they are betraying their bodies [41][37]. They experience a loss of control over their bodies for two reasons: the inability to control infertility and repeated medical interventions. These procedures intensify feelings of incompleteness and body fragmentation [42][19][43].

Depression is another significant trauma in RIF women that follows a non-linear pattern [44] (Figure-1D).

This means that initial hopefulness results in low depression at the start of treatment, but repeated treatment failures over the following years cause a significant rise in depression levels. However, after more than six years, the process of psychological adaptation to failures leads to a decrease in depression levels [45]. Findings indicate that the initial level of depression is four times higher in infertile women than in healthy women [46]. Researchers believe that infertility is not just a feeling of sadness, but also affects women's sense of self-attraction, raises anxiety, and reduces concentration [46][47]. Unlike, Lok et al. [48] reported that the severity of depression and the duration of infertility have a linear relationship. With treatment failure at the beginning of the study, psychological distress levels increased by roughly 10 percent, while depression levels remained unchanged. Additionally, a longer duration of infertility was linked to higher levels of depression [48]. This difference between Lok et al.'s findings and the nonlinear pattern results from variations in research methods, questionnaire types, and cultural factors [44]. Because many couples view infertility treatment as a major source of psychological stress and find existing supports inadequate, the importance of providing psychological services to those experiencing the highest levels of stress is emphasized [49][50].

RIF women face severe anxiety, stress, and depression, and it is reported that just one treatment failure can lead to some infertile women discontinuing treatment due to psychological distress [12][51]. Prescribing ovulation drugs and performing invasive surgeries can lead to mood changes, depression, fatigue, trouble sleeping, headaches, nausea, and pain for women receiving infertility treatment [52][53]. Furthermore, most women undergoing treatment report that these invasive procedures are humiliating [54]. However, repeated failures lead couples to turn to third-party options, such as surrogates or donated oocytes and sperm, which can cause significant psychological distress and stress in infertile women [55]. Psychological pressure and stress can be so intense that couples choose to stop treatment [19].

The impact of infertility on marital satisfaction varies among couples and depends on their individual beliefs, attitudes, and how couples handle infertility. For example, some religious beliefs view infertility as divine will, while some couples prefer adoption, which prevents them from undergoing infertility treatments [56][57][58]. During treatment, having sex on a scheduled basis can reduce intimacy and make it feel like a routine task. Because of this, the stress experienced by couples during treatment increases, and this can affect marital satisfaction either temporarily or in the long term [58][59][60][19].

Generally, sexual function in infertile couples is lower than in fertile couples [61]. Sexual dysfunction in RIF women is approximately 30% higher than in infertile women, which may be due to repeated treatment failures [62][63]. RIF women experience more pain during intercourse than infertile women who have not yet started treatment. Although the levels of sexual desire, arousal, lubrication, orgasm, and satisfaction are lower in RIF women than in infertile women [62][64]. Sexual performance dissatisfaction is lower in infertile women who had successful treatment compared to women with treatment failures and women who have adopted children [65]. Depression affects the sexual function of RIF women more negatively than anxiety and stress [66][67].

Financial pressures worsen the treatment burden of RIF patients. Because repeating cycles increase the costs of medication, tests, and invasive procedures, they expose the couple to tough decisions about whether to continue or stop treatment. Research indicates that in numerous countries, patients continue to experience significant challenges despite partial insurance coverage. Although financial pressures are an important factor in the decision to stop treatment, evidence indicates that the psychological and emotional burden of treatment is a more significant reason for discontinuation than financial costs. In other words, insurance can alleviate financial burdens, but it may not alleviate the psychological stress resulting from repeated failures and challenging treatment experiences [68][69][70].

In general, repeated failure creates a significant psychological burden on RIF women across emotional, social, familial, financial, and cultural aspects; therefore, offering more psychological services and support to these women seems essential [23][50].

## Effect of Psychological Factors on RIF

Screening for psychological disorders before ART treatment helps to better understand if the patient might experience mental or emotional challenges during and after infertility treatment [1]. Infertility is a chronic stressor because patients often face fertility problems for a long time before a clear diagnosis, and this experience causes stress [71][72]. There are two types of anxiety: a state in which a person is always anxious for no reason, known as trait anxiety. The anxiety that occurs temporarily due to infertility is known as state anxiety. Reports on the level of state anxiety are different at the beginning of ART treatment because of various cultures and attitudes [38]. Chen et al. [73] found that about 40% of infertile women had psychological disorders before starting treatment. Anxiety was more common than depression, which supports Verhaak et al.'s [38] findings on state anxiety.

Overall, only three studies on depression used accurate measurement tools before treatment, and their results were inconsistent; one found no difference between infertile patients and controls, while another showed that infertile patients scored higher on a depression scale before treatment compared to healthy controls [74][75][76]. Evidence shows that patients with a history of depression face a higher risk of recurrence during treatment. Diagnosing depression prior to treatment helps in managing depression during therapy [77].

Pretreatment Psychological distress is closely linked to treatment outcomes. These include medical diagnosis, number of treatment cycles, number of oocytes retrieved, fertility rate, number of embryos transferred, embryo quality, and confirm pregnancy [78]. In fact, the pretreatment distress level is negatively correlated with live birth rates [79][80].

Studies indicate that experiencing sexual abuse in childhood or adulthood, as well as domestic violence, is linked to a higher prevalence of gynecological problems and chronic pelvic pain in women [81][82]. Additionally, women with more positive expectations about motherhood and men who viewed the desire to have children as part of their sexual relationship had higher fertility compared to others [83][44].

Research evidence indicates that job stress may predict treatment failure in women [84]. Research indicates that certain lifestyle and psychosocial factors, such as psychological stress, negative life events, and high caffeine consumption, may be linked to fertility outcomes, although the evidence on their combined effects and conclusive impact remains limited [85][86]. Furthermore, excessive activation of cardiovascular responses to stress may be linked to decreased fertility [87].

## Psychoneuroendocrine Model for RIF

Hormones such as cortisol, prolactin, and insulin play a critical role in human reproductive function [88][89]. Cortisol secretion, the main stress hormone, increases via activation of the hypothalamic-pituitary-adrenal axis (HPA) and influences women's reproduction by suppressing immune responses [90]. The menstrual cycle regulates cortisol synthesis by modulating the HPA axis, so cortisol levels are lower in the follicular phase than in the luteal phase [91]. It is notable that cortisol levels can affect infertility and treatment failure, and these conditions can also influence cortisol levels [90] (Figure-2).

Studies indicate that cortisol levels are significantly higher in infertile women compared to fertile women [88][92][93][94]. Furthermore, pre-treatment cortisol levels are lower in infertile women who become pregnant at the end of treatment cycle compared to those who do not [88][94].

Cortisol negatively affects LH, FSH, progesterone and estradiol. When cortisol levels rise, it can disrupt ovarian function and the menstrual cycle. Cortisol affects on endometrial receptivity, reducing fertilization and pregnancy rate [90]. However, women with lower resilience have higher cortisol and stress levels [95]. Stress and age individually influence infertility, but they also have a combined effect. Aging impacts the HPA axis, which in turn affects cortisol regulation and the stress response level [96][90].

Furthermore, cortisol levels during the menstrual cycle of infertile women do not help predict treatment outcome. However, cortisol levels during anticipated stress before treatment are associated with greater stress-axis reactivity during treatment. Therefore, cortisol levels measured during anticipated stress before oocyte retrieval and embryo transfer are essential for predicting fertility outcomes [90][44].

The oxytocin axis influences the connection between psychological activities and female infertility. A positive mood and physical touch can raise oxytocin levels. Higher oxytocin is linked to lower rates of depression and plays a role in moving sperm through the female reproductive tract [44]. Oxytocin, through its receptors in the myometrium and endometrium of the uterus, can influence uterine contractions and factors linked to endometrial receptivity Since uterine contractions are negatively correlated with implantation, it has been suggested that increased activity of the oxytocin/receptor axis around embryo transfer could be one of the mechanisms affecting implantation failure and the failure of assisted reproductive treatments [97][98]. Blocking the oxytocin receptor has been associated with reduced uterine contractility, enhanced endometrial blood flow, and improved endometrial receptivity. Furthermore, administration of oxytocin receptor antagonists around the time of embryo transfer may increase the pregnancy outcome [97][98][99].

## Conclusion

Studies indicate that both physiological and psychological factors contribute to RIF. Implantation can be considered as a delicate process similar to a boat navigating changeable environmental conditions; stress and psychological disturbances may disrupt this balance and adversely impact treatment outcomes. More importantly, there are reciprocal relationships between psychological conditions and RIF. Nonetheless, medication therapy and hormonal interventions used to treat infertility may cause psychological symptoms observed in RIF women. Therefore, the psychological effects of the therapy process itself must be examined independently from the side effects of the drugs to appropriately measure the mental condition of the patients. Thus, the incorporation of psychological evaluation and support with medical interventions can significantly improve treatment outcomes and alleviate the emotional burden on RIF patients.

## Conflict of Interest

The authors have no relevant financial or non-financial interests to disclose.

## AI Disclosure Statement

AI tools were used solely for language improvement and editing purposes under direct human supervision. All substantive content, decisions, and final approval were made by the human author.
